# Workgroup Report: Incorporating *In Vitro* Alternative Methods for Developmental Neurotoxicity into International Hazard and Risk Assessment Strategies

**DOI:** 10.1289/ehp.9427

**Published:** 2007-02-06

**Authors:** Sandra Coecke, Alan M Goldberg, Sandra Allen, Leonora Buzanska, Gemma Calamandrei, Kevin Crofton, Lars Hareng, Thomas Hartung, Holger Knaut, Paul Honegger, Miriam Jacobs, Pamela Lein, Abby Li, William Mundy, David Owen, Steffen Schneider, Ellen Silbergeld, Torsten Reum, Tomas Trnovec, Florianne Monnet-Tschudi, Anna Bal-Price

**Affiliations:** 1 ECVAM—European Centre for the Validation of Alternative Methods, Institute for Health and Consumer Protection, European Commission, Joint Research Center, Ispra, Italy; 2 Johns Hopkins University, Center for Alternatives to Animal Testing (CAAT), Baltimore, Maryland, USA; 3 Syngenta CTL, Macclesfield, United Kingdom; 4 Polish Academy of Sciences, Warsaw, Poland; 5 Istituto Superiore di Sanità, Rome, Italy; 6 U.S. Environmental Protection Agency, Research Triangle Park, North Carolina, USA; 7 New York University School of Medicine, New York, New York, USA; 8 University of Lausanne, Lausanne, Switzerland; 9 Oregon Health & Science University, Portland, Oregon, USA; 10 Exponent, San Francisco, California, USA; 11 CEFIC—European Chemical Industry Council, Shell Chemicals Limited, London, United Kingdom; 12 BASF, Ludwigshafen, Germany; 13 Federal Institute for Drugs and Medical Devices, Bonn, Germany; 14 Slovak Medical University, Bratislava, Slovak Republic

**Keywords:** high-throughput screening, *in vitro* developmental neurotoxicity models, regulatory use, validation

## Abstract

This is the report of the first workshop on Incorporating *In Vitro* Alternative Methods for Developmental Neurotoxicity (DNT) Testing into International Hazard and Risk Assessment Strategies, held in Ispra, Italy, on 19–21 April 2005. The workshop was hosted by the European Centre for the Validation of Alternative Methods (ECVAM) and jointly organized by ECVAM, the European Chemical Industry Council, and the Johns Hopkins University Center for Alternatives to Animal Testing. The primary aim of the workshop was to identify and catalog potential methods that could be used to assess how data from *in vitro* alternative methods could help to predict and identify DNT hazards. Working groups focused on two different aspects: *a*) details on the science available in the field of DNT, including discussions on the models available to capture the critical DNT mechanisms and processes, and *b*) policy and strategy aspects to assess the integration of alternative methods in a regulatory framework. This report summarizes these discussions and details the recommendations and priorities for future work.

Chemicals present in the environment have a potential impact on neurodevelopment and children’s health. In recent years, much attention has been given to model development and risk assessment procedures for reproductive toxicity, but the specific area of developmental neurotoxicity (DNT) has been relatively neglected in testing and risk assessment studies. Although epidemiologic and animal studies on developmental neurotoxicants have been carried out (Evangelista de Duffard and Duffard 1996), most chemicals in use have been tested scarcely or not at all for DNT. To properly assess the risk of chemicals for human health, data on DNT are necessary and this need is recognized by all stakeholders.

In 1998, the U.S. Environmental Protection Agency (U.S. EPA 1998) published the Health Effects Test Guidelines OPPTS 8706300 on DNT (U.S. EPA 712-C-98-239), and the Organisation for Economic Co-operation and Development (OECD) is currently finalizing a new draft Test Guideline (TG) for DNT (OECD 2006). To support and promote these efforts, this workshop focused on two immediate needs for DNT testing: first, the identification of *in vitro* and nonmammalian alternative methods that may recapitulate critical aspects of the development of the human nervous system; and second, how results from such alternative methods could be integrated into current *in vivo* testing strategies and the existing regulatory framework. Our hope is that this approach will decrease the number of chemicals reliant on DNT data solely from *in vivo* mammalian DNT tests and, consequently, refine, reduce —and maybe partly replace—the need for animal testing. Furthermore, we hope this workshop report will provide the basis for discussion in the expert communities on DNT testing and that such a discussion will identify the best steps forward.

## Definition of DNT

Chemicals may adversely affect the nervous system in various ways (Ray 1999). They may perturb commitment of neural stem cells, proliferation of neuronal progenitor cells, cell migration, synaptogenesis, cell death, formation of transmitters and receptors, trimming of connections, myelinization, and development of the blood–brain barrier (BBB). Impairment of the nervous system can lead to a variety of health effects such as altered behavior, mental retardation, and other neurodevelopmental disabilities and diseases (Li et al. 2005; Olney 2002; Rodier 1995).

For the purpose of this report, DNT is defined as the adverse effects of substances (regulated foreign compounds or xenobiotics) on the nervous system associated with exposure during development. The adverse effects may be expressed at any time during the life span of the exposed individual.

## Available Tests Linked to DNT End Points, Processes, and Models

Alternative approaches to DNT testing can be divided into two classes: *in vitro* models and nonmammalian animal models. In the following section we summarize possible *in vitro* models for DNT testing and then elaborate in more detail on nonmammalian models for DNT testing, and conclude with a critical assessment of these approaches for DNT testing.

### In vitro *models for DNT testing.*

Many neural development processes are understood at a cellular and molecular level, and can partly be modelled *in vitro*. Cell culture techniques have been developed to address key biochemical and functional features of developmental neural biology such as cell migration, formation of neuronal networks, synaptogenesis, and neuron–glia interaction. Among others, these processes may be specifically targeted by developmental neurotoxicants *in vivo*. Therefore, the rationale for the use of *in vitro* models for DNT testing is based on a clear understanding of the mechanistic processes underlying normal nervous system development. However, each *in vitro* model has its own specific advantages and disadvantages (Tiffany-Castiglioni 2004) and represents different grades of complexity and allows predictions for humans to different degrees (Table 1).

In the following sections, we outline the potential uses of the less well-established but promising murine and human embryonic stem cells (ESC) and human neuronal stem cells (NSC) for DNT testing. For a brief discussion on the predictive capacity and inherent limitations of established *in vitro* models with potential use for DNT testing, see Tables 1 and 2 and the literature therein.

#### Rodent and human stem cells

Presently, the mouse embryonic stem cell test (EST) is the only system based on a mammalian cell line, which was successfully validated as an alternative for *in vivo* embryotoxicity testing (Genschow et al. 2002). Committing mouse ESCs into neuroectodermal fate or directing these cells to more advanced stages of neuronal development may extend the mouse EST to capture also DNT end points (Hareng et al. 2005). Similarly, human ESCs can be directed into all three major central nervous system (CNS) cell types, and the sensitivity of these cell types to compounds may be assessed (Zhang et al. 2001). However, human ES cell culture techniques still require optimization for DNT testing.

Another stem cell model for DNT testing might be somatic neural stem cell (sNSC) cultures. These cultures are characterized by their capacity to self-renew and to differentiate into neurons, astrocytes, and oligodendrocytes. These three cell types can interact with each other in two- and three-dimensional (neurosphere-like structures) cultures. This may provide the ability to assess the sensitivity of early and advanced human neural development to compounds by various means such as cell proliferation, cell migration, cell-type specific mRNA/protein expression, and electro-physiologic responses. Although sNSCs are still a fairly immature model, recent work on normal human neural progenitor cells and a nonimmortalized human cord blood–derived NSC line points to its potential for DNT testing (Buzanska et al. 2005; Fritsche et al. 2005).

### Alternative nonmammalian models for DNT testing

Current *in vivo* test methods for detecting neurotoxicity and DNT are based on a number of end points including behavioral tests that are considered by regulators as crucial for neurotoxicity risk assessment. *In vitro* models cannot recapitulate such complex end points. However, using nonmammalian species in alternative test strategies may help address some of these behavioral end points. Among nonmammalian model organisms, zebrafish and *C. elegans* are particularly suited to address neurotoxic and DNT end points. The ease of obtaining high numbers of progeny, the availability of neuronal tissue specific *in vivo* reporter strains and the inherent transparency of the embryos make these two model organisms amenable to high-throughput screening (HTS) (Peterson et al. 2000; Wittbrodt et al. 2002). Moreover, the basic understanding of gene function and physiology combined with well-characterized stereotypic behaviors provides the possibility of using zebrafish and *C. elegans* for neurotoxicity risk assessment based on behavioral end points (Orger et al. 2004). Additional nonmammalian species include the sea urchin (Buznikov et al. 2001; Cameron and Davidson 1991) and *Drosphila* (Grueber and Jan 2004; Jones et al. 2006); both are potentially useful systems, but details are not included here due to space limitations.

#### Medaka and zebrafish as potential models for DNT

Assays based on medaka and zebrafish measure general DNT end points such as cell proliferation, neuronal precursor differentiation, and maturation. The existing techniques allow the assessment of specific neuronal migration, axonal and dendritic outgrowths, pruning, synaptogenesis, development of neuronal circuits, and their ultimate function—behavior (Tables 2 and 3).

Neuronal precursor proliferation and molecular differentiation can be assayed *in vitro* and *in vivo. In vitro*, marker gene expression can be used to analyze the specification of major neuronal and glial cell types (neurons, oligodendrocytes, astrocytes, microglia) and neuronal subtypes (e.g., GABAergic neurons, glutaminergic neurons) (Brosamle and Halpern 2002). More recently, transgenic techniques allow the analysis of these marker genes in live animals by fluorescent protein expression (Higashijima et al. 2000; Park et al. 2000; Table 3).

Similarly, neuronal migration and morphologic differentiation can also be analyzed *in vitro* and *in vivo. In vitro*, a combination of marker gene expression and cell position can be used to assess neuronal migration. However, this is an indirect measure because incorrect patterning and morphogenesis of other tissues may affect neuronal migration and, thus, correct positioning. These caveats may, in part, be overcome by *in vivo* analyses of neuronal migration. Using transgenic techniques to fluorescently mark migrating cells, *in vivo* analyses of migration also offers the possibility to assess other cell dynamics besides correct cell positioning (Gilmour et al. 2002). Although more laborious, this allows motility and chemotaxis defects during neuronal migration to be distinguished, and may resolve secondary defects better than static *in vitro* analyses (Table 3).

Morphologic differentiation of neurons involves dynamic processes. Neurons extend axons and dendrites, establish and discontinue synaptic contacts, and mature into a neuronal circuit. *In vitro,* neuronal membrane–specific antibodies and, *in vivo,* membrane-tethered fluorescent proteins can be used to analyze these processes (Higashijima et al. 2000; Park et al. 2000).

Behavior is the ultimate effect of neuronal development. Certain behavioral responses of fish can be analyzed reasonably robustly, because some of the underlying neuronal circuits have been characterized (Baier 2000). Such behaviors may be classically analyzed by responses to certain stimuli or, more recently, molecularly by stimulus-induced gene expression.

#### Limitations of alternative models for DNT testing

The alternative approaches to DNT testing discussed above and in Tables 1–3 are potential models for DNT testing. To our knowledge none of these models have been used specifically for DNT testing up to this date. However, we believe that these models merit intensive consideration in drafting DNT testing strategies if one is aware of their limitations and caveats.

One concern is the predictive capacity of alternative models. How does one interpret an “effect” seen in an *in vitro* or nonmammalian model? Provided the molecular basis leading to the end point in question reflects *in vivo* development, an “effect” detected in an alternative model is a good indication that a similar effect may be expected in humans after chemical exposure. This is less of a concern when using nonmammalian models for DNT testing because mechanisms of neural development are highly conserved among distant species. On the other hand, interspecies differences may cause false positives or false negatives when screening compounds for adverse effects on humans, with nonmammalian models. The use of human cell systems will circumvent this problem. Therefore, a combination of nonmammalian and human cell–based models may maximize the predictiveness of alternative models.

Pharmacokinetics need to be considered when interpreting results from *in vitro* models. Because *in vitro* systems do not reflect *in vivo* absorption, distribution, metabolism, and excretion (ADME) of test compounds, results need to be interpreted with caution. This also holds true for nonmammalian *in vivo* models. These systems display ADME phenomena, but these phenomena do not necessarily reflect the human situation.

An additional, more practical concern is the amenability to automation and HTS. Depending on the model, the feasibility of HTS varies, and Tables 2 and 3 show to what extent we believe the different models were suited for such an approach.

As indicated above, some of the discussed caveats may be partly overcome by combining different alternative models in an intelligent testing strategy. Models with a well-characterized mechanism that reflects the *in vivo* situation, possibly based on human cell lines, in combination with nonmammalian models may more robustly detect compounds with adverse effects on humans. In addition, non-mammalian models may mimic human pharmacokinetics to a certain degree and offer the possibility to assess basic neuronal network functions such as simple behavior, although more complex behaviors relying on neocortical structures unique to mammals will escape detection. In this light, an intelligent combination of DNT tests may help refine the *in vivo* animal histology and behavioral testing battery used in the U.S. EPA and OECD guidelines.

## Systems Interaction and other Considerations

### Interaction/interplay between endocrine and immune function

Chemicals may interfere with or mimic the effects of endogenous hormones and signaling chemicals of the endocrine system. A well-known example is the interference of chemicals with the thyroid system (Colborn 2004; Damstra 2002). There are international efforts to validate or standardize screens or assays for detecting test chemicals with potential endocrine-disrupting effects. Among the chemicals on the market today, few if any have been systematically tested for such effects for regulatory purposes. These tests should be integrated in an overall hazard and risk assessment strategy for DNT.

### Importance of blood–brain barrier and choroid plexus in DNT

Alterations in both BBB and choroid plexus (CP) have been implicated in neurodevelopmental disorders. The integrity of the BBB and the CP barriers, both structurally and functionally, is essential for brain chemical stability. *In vitro* BBB and CP models (Prieto et al. 2004; Reichel et al. 2003) are available and can be used for evaluating the DNT potential of chemicals. The BBB is a special capillary bed that separates the blood from the CNS parenchyma. The CP produces the cerebrospinal fluid (CSF) and is involved in the most basic aspects of neural function including: maintaining the extracellular milieu of the brain by actively modulating chemical exchange between the CSF and the brain parenchyma, surveying the chemical and immunologic status of the brain, detoxifying the brain, secreting a nutritive mixture of polypeptides, and participating in repair processes after trauma (Emerich et al. 2005). Retrospective studies diagnosing human fetal anomalies of the CNS by ultrasonography and by fetal magnetic resonance imaging, combined with follow-up studies after birth, have revealed CP pathology in 9% of children with suboptimal neurodevelopmental (Leitner et al. 2004).

### Metabolism-mediated DNT effects

Biokinetic processes—such as absorption, distribution, biotransformation, and excretion—determine the relationship between the exposure pattern and the internal concentration time course. In addition to barrier effects, biotransformation may play a crucial role in DNT hazards. Biotransformation or metabolism is the process by which a substance in the body is chemically transformed to a metabolite or a variety of metabolites. Biotransformation is usually divided into two main phases, phase 1 and phase 2, the former of which is usually oxidative (e.g., hydrolysis, although reductive metabolism can occur) and predominantly catalysed by the many isoforms of the cytochrome P450 supergene family. Phase 2 is catalysed by a variety of enzymes that conjugate the oxidized moiety with highly polar molecules, such as glucose, sulphate, methionine, cysteine, or glutathione. The biokinetics of a compound, including its metabolism, can greatly influence its toxicologic properties. One of the most frequently cited limitations of nonhuman-based *in vitro* and *in vivo* assays is the qualitative and quantitative differences in the biotransformation of test chemicals, in comparison with human biotransformation (Coecke et al. 2006). The effect of human chemical biotransformation needs to be taken into account in both *in vivo* and *in vitro* assays. In the case of DNT, existing data indicate a role of biotransformation for DNT (Parmar et al. 2003). Estrogen formed in the brain and from other estrogen-synthesizing tissues is catalyzed by cytochrome P450 aromatase isoforms. Estrogen regulates neuronal, proliferation, survival, morphology, synaptogenesis, and differentiated functions in many various regions of the adult brain. Thus, inhibition or induction of these cytochrome P450 isoforms may cause alterations in these processes.

## Validation and Testing Strategies

To investigate chemicals that have the potential to cause DNT, we have illustrated the availability of *in vivo* and *in vitro* test methods. Both *in vitro* and nonmammalian test systems (particularly when used in combination) offer the possibility of providing an early screen for a large number of chemicals, and could be useful in characterizing the mechanism of action or the developmental processes that are particularly affected by the test chemical. *In vitro* assays may not always reflect the *in vivo* animal results because of species differences, absence of kinetic considerations, or a complex interplay between a diverse range of mechanisms and processes affected by the chemicals, including for example, the interaction/interplay between endocrine and immune function. Therefore, a battery of *in vitro* and *in vivo* assays seems at present the most appropriate way of providing the added value of the alternative approaches.

**Figure 1:**
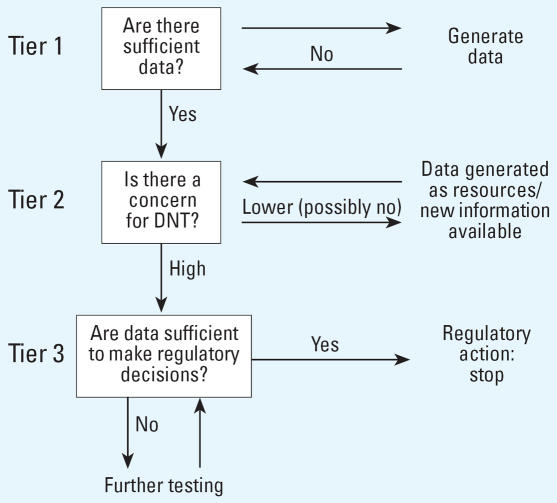
Scheme of the different steps in identifying DNT hazards. Two different levels were identified.

Any potential alternative test system must be validated and standardized before the information generated can be used for hazard identification and for risk assessment. Because adequate reference methods that reliably predict health effects are lacking and the *in vivo* approaches are complex, the assessment of the *in vitro* methods will not be a straightforward task. A significant problem with the existing *in vivo* methods for the identification of developmental neurotoxicants is the lack of explicit guidance on how to quantitate the risks of DNT [either for low observed effect level (LOEL) or no observed effect level (NOEL), or for benchmarks]. Moreover, it is difficult to interpret the methods in terms of their predictive value for human health. Figure 1 illustrates a strategy for toxicologic evaluation in the context of DNT testing, including the possible contribution of both *in vitro* and nonmammalian testing.

### Tier 1

Tier 1 incorporates existing knowledge including *a*) any animal studies, *b*) *in vitro* studies (cell and tissue cultures methods, conventional and novel end points such as “omics”), *c*) exposure information, *d*) epidemiology information, *e*) intended use, and *f* ) chemical structure and any relevant physicochemical data. The first step is a critical evaluation of the quality of existing information. If sufficient information is available at Tier 1, a decision can be made if there is a concern about DNT. If there are data gaps, new *in vivo* data should be generated and complemented by *in vitro* testing to enable a decision to be made regarding DNT potential. In cases where absolutely no chemical information exists, a base set of data should be generated [see OECD recommendations on Screening Information Data sets (SIDs) (International Programme on Chemical Safety 1996]. Both *in vitro* and nonmammalian test systems could be applied for initial screening to permit an approximate DNT assessment.

### Tier 2

In cases where data are available, a decision can be made whether there is a DNT concern (high or low priority). In cases where there is evidence for pre- and/or postnatal exposure in humans (e.g., detection of a chemical in breast milk), priority should be high. If information collected in Tier 1 is relevant to DNT, priority should be given for DNT testing. The evaluation of the exposure scenario will aid the decision as to whether the compounds are high or low priority for DNT testing, or if no further testing is required and a regulatory action can be taken. Well-conducted studies that indicate no current concern should move chemicals to the low-priority list for DNT testing. These compounds should be evaluated when new information becomes available from animal and alternative test methods. In cases where available data support a high concern for DNT, Tier 3 testing should be carried out.

### Tier 3

Based on the nature of the available data and regulatory requirements, this stage could include very specific tests, or it may involve the use of higher-order *in vitro* or nonmammalian alternative tests, or *in vivo* mammalian testing.

However, *in vitro* or nonmammalian alternative approaches may become important for this stage when the number of chemicals with no available data is very high. This is currently the case in different international regulatory environments. The drivers in Europe, for the research of alternative methods to replace conventional animal tests for toxicologic hazard assessments, are generally related to the chemicals and cosmetic regulations (Eskes and Zuang 2005; European Commission 2003). Also, with the High Production Volume Challenge Program in the United States (U.S. EPA 1998), a call for the replacement of animal experiments within a short-time frame is being launched. Therefore, Tier 3 in the *in vitro* alternative predictive test batteries strategy, based on end points, mechanisms, and processes relevant to DNT for chemical-induced neurotoxicity, might be of great added value and become increasingly more important for regulatory decision making. Tests may include the use of *in silico* tools in combination with one or more of the proposed alternative test systems targeting specific mechanistic or functional markers of developmental neurotoxicant-induced alterations. Such tests may use integrated genomic, proteomic, and other “omic” analyses and a variety of biochemical, morphologic, biotechnologic, or electrophysiologic profiling methods. Such a tiered testing scheme will allow more data to be generated in Tier 3 for those compounds where there is a concern for DNT, to allow regulatory decision making. Such tiered approaches that integrate batteries of *in vitro* alternative tests are currently proposed for ecotoxicologic risk assessment (Jeram et al. 2005).

## Conclusion

There is societal concern that the increasing prevalence of childhood behavioral diseases is related to augmented exposures to xenobiotics. Currently, there are thousands of chemicals that have not been evaluated for their potential to cause DNT. DNT tests used for regulatory decisions should predict and identify DNT hazards. Despite the empirical usefulness of *in vivo* animal tests, these tests consume a high number of animals, are labor intensive, complex in experimental design, not always sensitive enough, and often do not provide information that facilitates a detailed understanding of potential mechanisms of toxicity. Furthermore, *in vivo* mammalian tests are unsuitable for screening large numbers of agents, and their predictive capacity for the human situation remains unclear. Today, it would be too ambitious to pretend that an alternative DNT strategy would focus on replacement of the *in vivo* DNT test methods. A first step would be to refine the current *in vivo* strategies by integrating information derived from *in vitro* and nonmammalian alternative test strategies. *In vitro* DNT tests designed to identify chemicals with the potential to cause DNT would eliminate the number of chemicals reliant on DNT data solely from *in vivo* mammalian DNT tests, and therefore reduce animal testing. At the same time, primary neuronal cell cultures (organotypic, re-aggregating, or dissociated) are prepared from freshly isolated brain tissue, thus still consuming animals, although one animal provides material for many individual experiments. Because at present *in vivo*–based DNT testing cannot be replaced by *in vitro* approaches, incorporation of *in vitro* testing as a part of an intelligent testing strategy could at least refine and eventually reduce animal usage.

Combining *in vivo* data sets with *in vitro* approaches in intelligent test strategies is increasingly important for regulatory decision making. In addition, these approaches will also be the most efficient way to decrease costs and the amount of time required for testing. In this report we focused on the several processes involved in brain development and identified *in vitro* and nonmammalian tests that may allow the generation of data sets that can help identify DNT hazards. Although all the test systems described were not developed for regulatory purposes at this stage, if they prove useful, we hope that this report will encourage their further development to render them amenable to high-throughput approaches. In this context we consider a test system “useful” if it models a certain *in vivo* process and distinguishes known chemicals that interfere or do not interfere with this process. Thus, this report proposes an approach that would refine and potentially reduce the number of animal tests that need to be performed. Most important, test strategies/batteries are needed that can evaluate mechanisms responsible for DNT.

## Recommendations

- Focus on experimental designs relevant for regulatory methods.
  1. Catalog *in vitro* systems available in the research community (including academia and government), and explore how they can be developed for regulatory use.
  2. A reference list of potential DNT chemicals should be established that incorporates all available data. Benchmarking against experiences gained in other large international efforts (e.g., ReProTect; Hareng et al. 2005) is suggested.
  3. Proper experimental design of *in vitro* (and *in vivo*) DNT tests must have positive and negative controls. Therefore, there is an urgent need to generate high-quality data on chemicals with DNT potentials.
- Toward development of an integrated tiered approach.
  1. Further refine the tiered approach based on the integration of physicochemical data, and other data sets available for the *in vivo*/*in vitro* toxicologic effects; exposure use patterns, toxicokinetic, immunotoxicity, reproductive toxicity, endocrine disruption and available developmental toxicity data sets should be explored.
  2. Evaluate the introduction of an iterative tiered/battery approach for DNT testing that combines both *in vitro* and *in vivo* data sets.
- Increase knowledge of critical neurodevelopmental processes enabling comparisons of alternative DNT models.
  1. A strategy should be developed to evaluate reference chemicals in multiple models to identify those models to be considered for inclusion in an intelligent testing strategy.
  2. Evaluate, by literature searches, which end points and mechanisms are linked to adverse effects on the developing human nervous system.
  3. Catalog and correlate human developmental landmarks to findings/end points from animal and *in vitro* studies.
  4. Alternative approaches using contemporary human and rodent cell and tissue cultures and alternative species might be the way forward to achieve a greater understanding of the importance of considering species differences when evaluating the DNT hazards of xenobiotics. Until interspecies differences for DNT are better understood, use of both human and rodent cell and tissue cultures and alternative species should continue.
  5. Considerations should be given to metabolism-mediated toxic effects (inclusion of metabolic competence).
  6. With the recognition of the lack of knowledge in developmental biology and developmental neurotoxicology, we encourage exploration of the most fundamental research issues.
  7. A long-term goal should be to evaluate the use of “omics” in DNT assessment.

## Figures and Tables

**Table 1:** General characteristics of potential *in vitro* models for DNT testing.

Type of culture	Relevance for DNT	Main advantages	Main limitations
Organotypic cultures^a^	Derived from undifferentiated embryonic brain or spinal cord tissue (e.g., slices, explants) Develop into mature and interactive neuronal–glial tissue-like structure Used to study the mechanisms of morphologic and physiologic cell maturation that could be affected by toxicants	Presence of *in vivo*–like three-dimensional anatomic and functional organization such as tissue-specific cytoarchitecture, neuronal connectivity, electrophysiologic activity, complex glial–neuronal interactions	Low throughput Limited period of culture Possible necrosis in the tissue center due to the limited oxygen and nutrients supply
Re-aggregating brain cell culture^b^	Derived from dissociated embryonic brain cells that re-aggregate spontaneously under continuous gyratory agitation Reproduce 3D complexity, exhibiting a developmental pattern both morphologically and functionally similar to the original brain tissue *in vivo*	Presence of neuronal cell types corresponding to the original tissue Presence of all glial cell types, i.e., astrocytes, oligodendrocytes, microglia Glial cell proliferation and maturation, synaptogenesis, and myelination recapitulate *in vivo* development Formation of natural extracellular matrix Mature cultures exhibit spontaneous and evoked electrical activity Possibility to study microglial cell activation and astroglial reactivity as early markers of neurotoxicity Cultured in chemically defined medium The model is robust and provides large amount of material for multidisciplinary and multiparametric assays	Anatomic organization present in the original tissue is lost Most neurons are postmitotic at culture initiation Not suitable for studies at the single-cell level Variability between individual aggregates with respect to size, proportion of neurons versus glial cells, and electrical activity
Primary dissociated culture^c^	Used for mechanistic studies and characterization of endogenous factors that are crucial for the normal differentiation and function of the developing nervous system	Easy access to single-cell toxicity assay assessment Most of the neurodevelopmental features are preserved (cell death, glia progenitor proliferation, cell migration, synthesis of transmitters, and expression of their receptors or formation of neuronal connections) Possibility to use pure culture of each cell type or as mixed neuronal–glial culture to study their interaction Easy to obtain and maintain	Histotypic tissue organization is lost Isolated neurons are postmitotic Variability between neuronal–glial ratio
Immortalized human and rodent cell lines^d^	Derived from tumors or transformed cells (neuroblastomas, gliomas, and schwannoma cell lines) Suitable to study the mechanisms of cell differentiation because under the appropriate culture conditions (e.g., exposure to growth factors) the cells differentiate into non-dividing neuronlike cells, characterized by neurite outgrowth	Availability of human tissue Differentiated neuronlike cells express electrical activity, synthesis of various neurotransmitters, and expression of associated receptors and ion channels. Provide homogeneous cell populations in large quantities in a very reproducible manner	Because they are transformed cells, differentiation process may not be comparable to ”normal” cells, and their ultimate phenotype are often different from primary neurons Often neurites are not representative of either axons or dendrites and do not form functional synapses Usually only one cell type is present, cell–cell interaction is missing Genetic instability with increased number of passage

a Data on organotypic cultures from Braun et al. (2006); Chalisova et al. (2006); Chen et al. (2005); Ghoumari et al. (2005); Hechler et al. (2006); Heck et al. (2006); Neumann et al. (1996); Overstreet et al. (1997); Pinzon-Duarte et al. (2004); Stoppini et al. (1991); Strasser and Fischer (1995); Zimmer et al. (2000).

b Data on re-aggregating brain cell culture from Braissant et al. (2002); Braun et al. (2006); Eskes et al. (1999, 2002); Harry et al. (1998); Honegger (1985); Honegger and Monnet-Tschudi (2001); Honegger and Richelson (1979); Monnet-Tschudi et al. (1995a, 1995b, 1997b, 2000); Sales et al. (2004); Seeds and Vater (1971); Zurich et al. (2002, 2004).

c Data on primary dissociated culture from Bal-Price and Brown (2001); Demerens et al. (1996); Goldin et al. (2001); Howard et al. (2005); Krause et al. (2006); Lein et al. (1995); Sah and Matsumoto (1987); Sass et al. (2001); Veronesi (1992); van Pelt et al. (2005); Yamamoto et al. (2005).

d Data on immortalized human and rodent cell lines from Abdulla et al. (1995); Greene (1978); Hong et al. (2003); Pahlaman et al. (1990); Parran et al. (2001); Sachana et al. (2001).

**Table 2:** Overview of promising *in vitro* alternative models^a^ and their characteristics.

*In vitro* models/processes^b^	Human stem cell/precursor cells ###	Rodent stem cells ###	Immortalized human cell lines neuronal/nonneuronal #	Immortalized rodent cell lines neuronal/nonneuronal #	Brain aggregates ###	Primary dissociated cultures ##	Organotypic cultures ##
Cell proliferation	++	++	++	++	++ (glial only)	++ (glial only)	—
	Li et al. 2005 Zhang et al. 2001	Milosevic et al. 2005	Shastry et al. 2001	Margioris et al. 1995	Honegger and Richelson 1979	Kinsner et al. 2005	
Precursor cell differentiation	++	++	++	++	++ (glial only)	—	+
	Carpenter et al. 2001	Takahashi et al. 1999	Shastry et al. 2001	Greene1978	Guentert-Lauber and Honegger 1985		Pinzon-Duarte et al. 2004
Glial reactivity	+	+	—	++	++	++	+
	Turka et al. 1995	Martinez-Contreras et al. 2002		Seidman et al. 1997	Monnet-Tschudi et al. 1995a, 1995b; Zurich et al. 2002	Bal-Price and Brown 2001	Neumann et al. 1996
Glial maturation (myelination)	+	+	++	++	++	++	+
	Windrem et al. 2004	Brüstle et al. 1999	Liu et al. 2000 Peden et al. 1990	Garcia et al. 2001	Honegger and Matthieu 1985	Demerens et al. 1996	Ghoumari et al. 2005
Migration	+/−	+/−	—	—	+	++	++
	Imitola et al. 2004	Imitola et al. 2004			Levitt et al. 1976	Sass et al. 2001	Heck et al. 2006
Axon/dendritic outgrowth	+	++			++	++	++
	Harper et al. 2004	Yoon et al. 2005	Abdulla et al. 1995	Parran et al. 2001	Braissant et al. 2002	Howard et al. 2005	Hechler et al. 2006
Apoptosis	++	++	++	++	++	++	++
	Li et al. 2005	Milosevic et al. 2005	Ba et al. 2003	Pittman et al. 1993	Monnet-Tschudi 1998	Dessi et al. 1995	Chalisova et al. 2006
Synapse formation	+	++	—	—	++	++	++
	Cummings et al. 2005	Copi et al. 2005			Seeds and Vater 1971; Monnet-Tschudi et al. 1995b	Yamamoto et al. 2005	Chen et al. 2005
Synapse pruning			—	—	—	++	+
						Goldin et al. 2001	Overstreet et al. 1997
Neurotransmitter receptor profiles	+	++	++	++	++	++	++
	Zhang et al. 2001; Carpenter et al. 2001	Ma et al. 2004	Lambert and Nahorski 1990	Whiting et al. 1987	Honegger and Richelson 1979	Sah and Matsumoto 1987	Zimmer et al. 2000
Neuronal connectivity	+	++	—	—	—	++	—
	Benninger et al. 2003	Toda et al. 2000				van Pelt et al. 2005	

a Grading of technical feasibility and accessibility of *in vitro* alternative models available: ###, complex; ##, less complex; #, simple.

b Grading of availability and extend of literature related to the processes for a specific alternative model: ++, currently available; +, potential; —, not yet available. Some of these systems are high-throughput, whereas others have the potential to be developed.

**Table 3:** Overview of promising nonmammalian alternative models^a^ and their characteristics.

Nonmammalian models/processes^b^	Zebrafish ##	Medaka ##	*C. elegans* #
Cell proliferation	++	++	++
	Wullimann and Knipp 2000	Candal et al. 2005	van den Heuvel 2005
Precursor cell differentiation	++	++	++
	Bertrand et al. 2002	Hirose et al. 2004	Chisholm and Jin 2005
Glial reactivity	—	—	—
Glial maturation (myelination)	+	—	—
	Brosamle and Halpern 2002		
Migration	++	++	++
	Gilmour et al. 2002	Hirose et al. 2004	Hatten 2002
Axon/dendritic outgrowth	++	++	++
	Beattie et al. 2002	Ishikawa et al. 2004	Colamarino and Tessier-Lavinge 1995; Cooper 2002
Apoptosis	++	++	++
	Cole et al. 2001	Stokes et al. 2004	Lettre and Hengartner 2006
Synapse formation	++	—	++
	Hutson and Chien 2002		Ackley and Jin 2004; Jin 2002
Synapse pruning	++	—	++
	Hutson and Chien 2002		Wiggin et al. 2005
Neurotransmitter receptor profiles	++	++	++
	Higashijima et al. 2004	Hamm et al. 2001	Komuniecki et al. 2004
Neuronal connectivity	++	—	++
	Godinho et al. 2005		Volovitch et al. 1993

a Grading of technical feasibility and accessibility of *in vitro* nonmammalian models available: ###, complex; ##, less complex; #, simple.

b Grading of availability and extent of literature related to the processes for a specific alternative model: ++, currently available; +, potentially available; —, not yet available. Some of these systems are amenable for high-throughput screening.
